# Short-Term Assessment of Cention N vs. Glass Ionomer Cement (Fuji IX) as a Definitive Restoration in the Primary Dentition of Mexican Children: A Pilot Study

**DOI:** 10.7759/cureus.83784

**Published:** 2025-05-09

**Authors:** Cecilia A Padilla-Ocampo, Daniel Medecigo-Costeira, José R Pioquinto-Mendoza, José de Jesús Navarrete-Hernández, Martha Mendoza-Rodríguez, María de L Márquez-Corona, Juan J Villalobos-Rodelo, Juan F Casanova-Rosado, Carlo E Medina-Solís, Gerardo Maupomé

**Affiliations:** 1 Academic Area of Dentistry of the Health Sciences Institute, Autonomous University of the State of Hidalgo, Pachuca, MEX; 2 Dentistry Department, Children's Hospital DIF Hidalgo, Pachuca, MEX; 3 Research and Teaching Department, Children's Hospital DIF Hidalgo, Pachuca, MEX; 4 School of Dentistry, Autonomous University of Sinaloa, Culiacan, MEX; 5 School of Dentistry, Autonomous University of Campeche, Campeche, MEX; 6 Advanced Studies and Research Center in Dentistry "Dr. Keisaburo Miyata" School of Dentistry, Autonomous University of the State of Mexico, Toluca, MEX; 7 Richard M. Fairbanks School of Public Health, Indiana University, Indianapolis, USA

**Keywords:** alkasite, arm, glass ionomer, oral health, pediatric dentistry

## Abstract

Background

Dental caries in primary dentition requires restorative materials that combine durability, strength, and therapeutic properties. Glass ionomer cements (GICs) are widely used, but their lack of stability sometimes limits their application. Cention N (Ivoclar Vivadent, Schaan, Liechtenstein), an alkasite restorative material (ARM), is an attractive option due to its greater mechanical strength and ion-releasing capacity compared to GIC. The objective of this study was to compare the clinical efficacy of Cention N versus a high-viscosity GIC (Fuji IX, GC Corporation, Tokyo, Japan) in primary tooth restorations, evaluating retention, fracture, and marginal color stability for the first six months after placement.

Methods

A quasi-experimental study was conducted in 16 patients (3-9 years old) with 36 restorations (Classes I, II, and V) in a public hospital in Mexico. Materials were assigned according to clinical criteria, ARM (n=28), with universal adhesive and light-curing, and GIC (n=8), following standard protocols. The variables evaluated were retention, fracture, and marginal color change, using monthly visual/tactile inspection. Statistical analysis employed frequencies, percentages, and Fisher's exact test (Stata Statistical Software: Release 14 (2015; StataCorp LLC, College Station, Texas, United States)).

Results

The mean age of participants was 7.29±1.53 years, ranging from three to nine years. The majority were male (62.5%). More ARM restorations were placed (n=28; 77.8%). Most cavities were Class V (30.6%). Regarding the clinical characteristics evaluated, fracture was observed in 8.3% of the restorations, color change occurred in 5.6%, and dislodgement occurred in 19.4%. Regarding the basal analysis of tooth type, number of restorations per tooth, sex, and age according to the type of restorative material used, no significant differences (p>0.05) were found. There were no significant differences in marginal color change (p>0.05). Regarding retention, ARM showed a significantly lower dislodgement rate (3.6%, 1/28) compared to GIC (75%, 6/8; p=0.000; Fisher's exact test). Regarding fracture resistance, no restorations with ARM presented fractures (0%, 0/28), while GIC recorded 37.5% (3/8) of fractures (p=0.008).

Conclusion

ARM showed better clinical performance in retention and structural integrity compared to GIC, without compromising margin aesthetics. Its mechanical properties make it a viable alternative for restorations in primary dentition. Longer-term studies are necessary to ascertain performance over a period of time that is closer to the expected restoration longevity for GIC and ARM.

## Introduction

Oral health is a fundamental component of general well-being, serving as the foundation for dental health across the lifespan and significantly influencing individuals' physical and psychological development [1]. From an epidemiological perspective, dental caries persists as the most prevalent chronic non-communicable disease globally, with a disproportionate incidence in vulnerable pediatric populations, affecting both primary and permanent dentitions and exerting a considerable burden on individuals, families, and public health systems [2,3]. Its etiology is multifactorial, where microbiological (biofilm imbalance), socioeconomic (limited access to prevention), and behavioral (hygiene habits) factors interact. In children, untreated caries can lead to chronic pain, infections, nutritional deficiencies, abnormalities in eruption, and disruptions in craniofacial development [3-5].

Restorative materials commonly used for carious lesions include amalgam, composites, and glass ionomer cements (GICs), each offering distinct characteristics and limitations. Selecting an appropriate material is crucial for ensuring the durability and functionality of restorations, particularly in primary teeth, where unique biomechanical factors and patient cooperation challenges must be considered [6,7]. GICs have been widely adopted in pediatric dentistry due to their chemical adhesion to dental tissues, biocompatibility, and capacity to release fluoride, which promotes dental tissue remineralization [8-10]. Their ability to chemically bond to enamel and dentin without complex adhesive systems makes them especially valuable in pediatric patients, where cooperation and chairside time are often limited [9,11]. Additionally, fluoride release from GICs provides an anticariogenic effect, helping to prevent recurrent caries at restoration margins and adjacent surfaces, making them ideal for managing primary teeth where caries control is critical [9,12]. Despite these advantages, GICs present notable limitations, particularly regarding mechanical properties. Their low fracture resistance and fragility restrict their use in restorations exposed to high occlusal loads, such as Class I and II cavities in primary molars [13,14]. Clinical studies [15-17] have demonstrated that while GICs perform adequately in small cavities not subject to intense masticatory forces, their longevity in larger restorations is questionable due to wear, fracture, and marginal integrity loss over time. These limitations are especially evident in pediatric patients with parafunctional habits such as bruxism or in teeth with substantial tooth loss, prompting the search for alternative materials that retain the therapeutic benefits of GICs while offering improved mechanical performance [9,10,18].

Cention N (Ivoclar Vivadent, Schaan, Liechtenstein), introduced in 2016, represents one such alternative. Classified as an alkasite restorative material, Cention N combines desirable mechanical properties with the release of fluoride, calcium, and hydroxide ions [19-24]. It features an alkaline filler system (Isofiller) designed to reduce polymerization shrinkage, a major factor associated with microleakage [19-24]. Clinically, Cention N offers versatility, as it can be used with or without conventional adhesive systems due to its self-etching mechanism, an advantage in pediatric dentistry where procedural simplification is highly beneficial [19,20,24]. Although in vitro studies have reported benefits such as reduced polymerization shrinkage and improved color stability compared to traditional GICs [25,26], these findings are largely based on laboratory experiments. Recent randomized controlled trials have demonstrated the efficacy of alkasite materials in primary molars. Specifically, Bhat et al. [27] reported superior performance of Cention N in Class II restorations compared to resin-modified GIC, with 92% retention rates at 12 months, supporting its potential as an alternative to conventional materials in pediatric dentistry. Clinical evidence regarding its performance in primary teeth, particularly among populations with high caries prevalence, remains scarce. Preliminary data suggest potential advantages, but further longitudinal clinical studies are necessary to validate its effectiveness under real-world conditions [19,20,24].

The objective of this pilot study was to clinically evaluate the efficacy of ARM compared to GIC as a definitive restorative material in primary dentition, assessing parameters such as retention, fracture resistance, and marginal color stability over a six-month follow-up period.

## Materials and methods

Study design

A comparative quasi-experimental study with a six-month follow-up was conducted to evaluate the clinical performance of Cention N versus high-viscosity GIC (Fuji IX, GC Corporation, Tokyo, Japan) in primary tooth restorations. The design included non-randomized groups. The present study was not funded by industry.

Population and sample

The target population consisted of pediatric patients (3-9 years of age) seen at the Pediatric Dentistry Clinic of the Children's Hospital DIF Hidalgo (Pachuca, Mexico) between July 2023 and February 2024. Using convenience sampling (a non-probabilistic method), 16 volunteer patients requiring 36 restorations in primary teeth were enrolled in the study. The inclusion criteria considered patients from the Pediatric Dentistry Clinic of the hospital between three and nine years of age, of either sex, with primary teeth requiring restorations of Class I, II, and V cavities, and whose parent or guardian signed the consent. The exclusion criteria were teeth with pathology and/or pulp exposure, uncooperative patients where treatment could not be performed, and patients with inadequate oral hygiene. Oral hygiene was assessed using the O'Leary plaque index (1% erythrosine), using a plaque disclosing agent. The smooth surfaces (buccal, lingual/palatal) of all teeth were examined, excluding the occlusal surfaces. The percentage of plaque was calculated by dividing the number of stained surfaces by the total number of surfaces evaluated and multiplying by 100. Good oral hygiene was considered adequate when the result was less than 20%.

Study variables

Dependent variables were dislodgement (total loss of restoration), fracture incidence (no/yes), and marginal color stability (assessed visually and with a colorimeter). The principal independent variable was restorative material (ARM vs. GIC).

Clinical procedures (interventions)

ARM Placement Protocol (per Manufacturer Specifications)

Achieve absolute or relative isolation of the tooth surface as needed. Remove carious tissue from interproximal, cervical, or occlusal surfaces (for interproximal restorations, after cavity preparation, place a metal matrix band to establish proximal contour). Apply 2.25% sodium hypochlorite (one minute contact time). Rinse thoroughly for 10 seconds and dry. Etch with 37% phosphoric acid (Scotchbond™ universal etchant, 3M, Saint Paul, Minnesota, United States) for 20 seconds. Rinse copiously with water for 20 seconds. Dry the cavity and tooth surface completely. Apply universal adhesive to enamel and dentin (20 seconds), and then air-thin gently for five seconds. Mix ARM powder and liquid at a 1:1 ratio. Bulk-fill the prepared cavity with ARM. Anatomically contour the primary tooth morphology. Light-cure for 20-30 seconds using LED curing light (operator-determined duration). Perform final occlusal adjustment and evaluation.

GIC (Fuji IX) Placement Protocol (per Manufacturer Specifications)

Isolate the tooth absolutely or relatively as needed. Excavate carious tissue from interproximal, cervical, or occlusal surfaces (for interproximal restorations, place a celluloid matrix band after cavity preparation). Apply 2.25% sodium hypochlorite (one minute application time). Rinse thoroughly for 10 seconds. Condition the cavity with GIC liquid conditioner. Prepare the material at a 1:1 powder-to-liquid ratio. Bulk-fill the cavity with the mixed GIC. Anatomically contour to restore primary tooth morphology. Verify and adjust occlusion.

Evaluation and statistical analysis

The restorations were evaluated monthly for six months. To ensure the objectivity and reproducibility of the evaluations, a standardized protocol was implemented that combined visual/tactile inspection, occlusion tests, and standardized photographs. The restorations were evaluated by visual and tactile inspection with a sterile dental mirror and WHO probe under artificial lighting type D (daylight), recording the findings according to standardized criteria by a single evaluator. Additionally, clinical photographs were captured with a high-resolution digital camera (100 mm macro lens and circular flash) including a VITA® shade scale (VITA Zahnfabrik, Bad Säckingen, Germany) in each image to calibrate the white balance and quantify clinically relevant color changes. Inter-examiner agreement was ensured through a prior calibration protocol, in which the evaluator performed 20 pilot evaluations under controlled conditions. During the study, consistency was monitored by re-evaluating these same pilot cases. To validate shade discrimination, a specific test was applied that required matching pairs of shades from the VITA® classical guide (shade A1-D4, VITA Zahnfabrik, Bad Säckingen, Germany) within 10 minutes, achieving a Cohen's kappa coefficient of >0.85. This test was repeated every four patients to maintain reliability. This multicomponent approach minimized bias and strengthened internal validity, a critical aspect given the limited sample size.

Statistical analysis was conducted using Stata Statistical Software: Release 14 (2015; StataCorp LLC, College Station, Texas, United States). Frequencies and percentages were used for qualitative variables, while means and standard deviations were calculated for quantitative variables. Bivariate analyses were performed using Fisher's exact test, chi-squared test, and the Mann-Whitney U test.

A post-hoc power analysis was conducted using G*Power (Version 3.1.9.7, Heinrich-Heine-Universität Düsseldorf, Düsseldorf, Germany) [28], which demonstrated that despite the limited sample size (n=36), the observed effect sizes were sufficiently large to achieve statistical significance (p<0.05) for all primary outcomes. This is reflected in the high noncentrality parameter (λ), which indicates that the between-group differences (e.g., 3.6% vs. 75% for eviction and 0% vs. 37.5% for fractures) were clinically and statistically relevant. This approach is supported by the methodological literature, which recognizes that large effects can be detected even with small samples when λ is high [29,30].

Ethical considerations

The study received approval from the Institutional Review Board of Children's Hospital DIF Hidalgo (approval number: CICEICB-EOP-2023-01), in compliance with the principles outlined in the Declaration of Helsinki. Written informed consent was obtained from all legal guardians, and strict measures were implemented to ensure data confidentiality throughout the research process.

## Results

The study evaluated 36 restorations in 16 patients. The mean age of participants was 7.29±1.53 years, ranging from three to nine years. The majority were male (62.5%) (Table 1). Table 1 also presents the basal analysis of tooth type, number of restorations per tooth, sex, and age according to the type of restorative material used, where no significant differences (p>0.05) were found. The descriptive results are in Table 2. More ARM restorations were placed (77.8%). Most cavities were Class V (30.6%). Regarding the clinical characteristics evaluated, dislodgement occurred in seven (19.4%) restorations, fracture was observed in three (8.3%), and color change occurred in two (5.6%). 

**Table 1:** Baseline characteristics of the study participants *: chi-squared test; †: Mann-Whitney U test SD: standard deviation; ARM: alkasite restorative material (Cention N); GIC: glass ionomer cement (Fuji IX)

	Frequency (%)	ARM group n (%)	GIC group n (%)	Statistic values	P-value
Sex					
Girls	6 (37.5)	11 (78.6)	3 (21.4)	X^2^=0.0083	0.927*
Boys	10 (62.5)	17 (77.3)	5 (22.7)		
Tooth type					
Anterior	9 (25)	5 (55.6)	4 (44.4)	X^2^=3.4286	0.064*
Posterior	27 (75)	23 (85.2)	4 (14.8)		
Age (mean±SD)	7.29±1.53	7.17±1.56	7.44±1.70	z=-0.774	0.4392†
Restorations per child	2.25±1.84	2.21±1.96	2.5±0.70	z= -1.012	0.3117†

**Table 2:** Description of the study variables regarding performed treatments (n=36)

	Frequency (%)
Treatment	
Cention N	28 (77.8)
Glass ionomer cement (Fuji IX)	8 (22.2)
Therapeutic cavity	
Class V (cervical)	11 (30.6)
Class I (occlusal)	6 (16.7)
Class II (occluso-mesial)	9 (25)
Class II (occluso-distal)	10 (27.8)
Restoration retention	
Retention: optimal	29 (80.6)
Retention: failed	7 (19.4)
Fracture	
Fracture: no	33 (91.7)
Fracture: yes	3 (8.3)
Marginal color stability	
Color stability: maintained	34 (94.4)
Color stability: compromised	2 (5.6)

Tables 3-5 show the bivariate analysis of the evaluated material characteristics. Regarding retention (dislodgement), ARM showed a significantly lower dislodgement rate (3.6%) compared to GIC (75%) (Fisher's exact test; p<0.001) (Table 3). Regarding fracture resistance, no restorations with ARM presented fractures (0%), while GIC recorded 3/8 (37.5%) fractures (Fisher's exact test; p=0.008) (Table 4). Finally, in marginal color stability, no significant differences were observed between materials (p=0.400), and ARM presented 1/28 (3.6%) marginal color changes, compared to 1/8 (12.5%) of GIC (Table 5).

**Table 3:** Restorative material comparison: dislodgement frequency Fisher's exact test: p<0.001 (for dislodgement comparison between materials)

Treatment	No dislodgement n (%)	Dislodgement n (%)	Total
Cention N	27 (96.4)	1 (3.6)	28 (100)
Glass ionomer cement	2 (25)	6 (75)	8 (100)
Total	29	7	36

**Table 4:** Fracture incidence by restorative material Fisher's exact test: p<0.01 (for fracture incidence comparison between materials)

Treatment	No fracture n (%)	Fracture n (%)	Total
Cention N	28 (100)	0 (0)	28 (100)
Glass ionomer cement	5 (62.5)	3 (37.5)	8 (100)
Total	33	3	36

**Table 5:** Marginal discoloration by restorative material Fisher's exact test: p>0.05 (for marginal discoloration comparison between materials)

Treatment	No marginal discoloration n (%)	Marginal discoloration n (%)	Total
Cention N	27 (96.4)	1 (3.6)	28 (100)
Glass ionomer cement	7 (87.6)	1 (12.5)	8 (100)
Total	34	2	36

## Discussion

This study evaluated the clinical efficacy of ARM (Cention N) compared to GIC (Fuji IX) as a restorative material for primary dentition. The results demonstrated that ARM outperformed GIC in terms of retention and fracture resistance, with no significant differences observed in marginal color stability. These findings suggest that ARM is a viable alternative for pediatric dental care. It is important to note that the clinical success of dental restorations depends on multiple factors, including the pediatric patient's cooperation during treatment, primary tooth characteristics, oral hygiene practices, the presence of biofilm, and the appropriate selection of restorative materials [31].

The significantly lower dislodgement rate (3.6% vs. 75%) and absence of fractures in ARM restorations (0% vs. 37.5% for GIC) can be attributed to its superior mechanical properties. Previous studies [19,20] have emphasized that ARM's alkaline composition, combined with its alkaline filler system, helps reduce polymerization shrinkage and enhances adhesion, supporting greater durability in Class I and II cavities. These findings align with the results reported by Arora et al. [32], who observed excellent retention of Cention N restorations after a nine-month follow-up. The longevity of direct restorations in the posterior region heavily relies on compressive strength, a key mechanical property; materials with low compressive strength, like natural tooth structure, are prone to fracture under occlusal forces [32].

Although no statistically significant differences were observed regarding marginal color stability (p=0.400), the low incidence of marginal discoloration in ARM restorations (3.6%) suggests good short-term adaptation and sealing. This outcome may be explained by ARM's self-etching adhesion mechanism and controlled ion release, which together reduce microleakage. Ballal et al. [33] similarly found that ARM maintained acceptable aesthetics in cervical restorations over medium-term evaluations. Nonetheless, it is important to recognize that longer follow-up periods could reveal differences not apparent within the six-month timeframe of this study. Equally, our results align with recent clinical evidence. Bhat et al. [27] similarly found that ARM outperformed GIC derivatives in retention (92% vs. 68%) and fracture resistance in Class II primary molar restorations; despite differences in follow-up duration, this consistency across populations reinforces the material's reliability.

Future research should involve extended follow-up periods (12-24 months), larger sample sizes, and multicenter study designs to validate these findings across diverse populations. Studies by Derchi et al. [34] and da Cunha et al. [35] have initiated the exploration of ARM's long-term performance in primary dentition, but further evidence is needed to solidify its clinical role. This study contributes to the evidence base supporting restorative decision-making in pediatric dentistry, aiming to optimize clinical outcomes.

Overall, the results support ARM as a promising restorative material for primary dentition, offering superior mechanical properties, therapeutic ion release, and clinical versatility. Its use is particularly advantageous in cavities exposed to high occlusal forces, where the fragility of traditional GICs limits their applicability. Moreover, this study provides the first clinical evidence in a Mexican pediatric population with high caries experience, suggesting that ARM could serve as a more durable alternative for restorations subjected to functional stress, particularly in public dental health programs. Although longer-term studies are needed to confirm these findings, the present research pointed to key parameters for future investigations.

Limitations of the study

This study presents several limitations that should be considered when interpreting the results. First, material allocation was based on clinical criteria rather than randomization, introducing the potential for selection bias. Additionally, the unequal distribution of groups, with a smaller sample size for the GIC group, may limit the statistical comparability and generalizability of the findings. In total, only 16 patients and 36 restorations were included, with just eight restorations using GIC, reducing statistical power and the ability to detect significant differences in variables such as marginal color stability (p>0.05). Nevertheless, despite the limited sample size, particularly for GIC, the large effect sizes observed (e.g., a 71.4% absolute difference in dislodgement rates) resulted in a high non-centrality parameter (λ), allowing for the detection of statistically significant differences.

Furthermore, although key clinical parameters were assessed, the follow-up period was short. Longer follow-up (12-24 months) is recommended to assess the long-term durability of both materials, especially in the primary dentition under functional loads. Marginal color stability was evaluated through visual inspection and a basic colorimeter, without the use of standardized spectrophotometric systems (e.g., CIELAB/CIEDE2000 standards using SpectroShade®, VITA Easyshade®, or Crystaleye® devices), which may limit sensitivity to detect subtle changes (ΔE>2.0) and introduce inter-examiner variability. Additionally, evaluators were not blinded to the material used, which could influence the interpretation of results.

While these limitations do not invalidate the findings, they underscore the need for further research to generate more robust evidence regarding the clinical performance of ARM in pediatric dental care.

## Conclusions

This short-term study showed that the alkasite restorative material Cention N works better than GIC for primary teeth. It has better retention, is more resistant to fractures, and maintains color stability at the edges. After six months of follow-up, ARM restorations showed a much lower rate of dislodgement and fewer fractures compared to GIC, which had higher failure rates for both measures. These results suggest that ARM could be a more durable and stronger option, particularly in cavities that face high biting forces, like Class I and II cavities, where the mechanical limits of GIC might affect long-term success. Furthermore, the low incidence of marginal color changes with both materials indicates that acceptable clinical aesthetics are maintained in the short term. While the findings are promising and reinforce the potential use of ARM in pediatric dentistry, research with larger sample sizes and extended observation periods is needed to confirm its long-term efficacy and durability in diverse populations.
